# Identification of biomarkers for tumor regression grade in esophageal squamous cell carcinoma patients after neoadjuvant chemoradiotherapy

**DOI:** 10.3389/fonc.2024.1426592

**Published:** 2025-01-17

**Authors:** Zhifu Chen, Yan Wang, Jun Chen, Zijun Xu, Tingjuan Zhang, Lu Sun, Lihua Zhu, Liben Xu, Chaoyang Wu, Zhiyuan Qiu, Dianjun Wang, Ting Wu

**Affiliations:** ^1^ Department of Radiation Oncology, The People's Hospital Affiliated to Jiangsu University, Zhenjiang, Jiangsu, China; ^2^ Central Laboratory, The People's Hospital Affiliated to Jiangsu University, Zhenjiang, Jiangsu, China; ^3^ Department of Oncology, The People's Hospital Affiliated to Jiangsu University, Zhenjiang, Jiangsu, China; ^4^ Department of Pathology, The People's Hospital Affiliated to Jiangsu University, Zhenjiang, Jiangsu, China

**Keywords:** esophageal squamous cell carcinoma (ESCC), neoadjuvant chemoradiotherapy (NCRT), tumor regression grade (TRG), GPR56, prognosis

## Abstract

**Background:**

Esophageal cancer is a highly invasive malignancy. Neoadjuvant chemoradiotherapy not only increases the rate of complete resection but also improves the median survival. However, a sensitive biomarker is urgently needed in clinical practice.

**Methods:**

60 esophageal squamous cell carcinoma (ESCC) patients undergoing neoadjuvant chemoradiotherapy (NCRT) were enrolled at the People's Hospital Affiliated to Jiangsu University. Patients were grouped according to tumor regression grade (TRG) criteria from the College of American Pathologists (CAP). The correlation between TRG groups, clinicopathologic characteristics, and prognosis was analyzed. Differential gene expression analysis was performed on ESCC patients before and after NCRT using the public database (GSE43519). MMP9, NFIX, and GPR56 were identified as candidate genes, and their expression and correlation with prognosis were evaluated by immunohistochemical analysis.

**Results:**

Among 60 ESCC patients who underwent surgery after NCRT, the pathological complete response (pCR) rate was 35.0% (21/60), and the major pathological response (MPR) rate was 60.0% (36/60). Poor tumor differentiation and neural or vascular invasion were associated with inadequate tumor regression grade and were independent factors influencing TRG. ESCC patients were divided into effective (TRG 0 + 1) and ineffective (TRG 2 + 3) groups. Higher TRG was significantly associated with shorter overall survival (OS). Our study also identified TRG as an independent prognostic factor through univariate and multivariate Cox regression analyses (*P* < 0.05). The differentially expressed genes GPR56, MMP9, and NFIX selected from the GSE43519 dataset were significantly downregulated after NCRT (*P* < 0.001). Immunohistochemistry showed that GPR56 was highly expressed in ESCC, while it was negatively expressed in paracancerous tissues. There was a significant difference in expression between cancerous and paracancerous tissues. GPR56 expression was consistent with the public dataset, and patients with high GPR56 expression had significantly shorter OS (*P* < 0.05). In addition, patients with inadequate MPR and high GPR56 expression had shorter OS (*P* < 0.05).

**Conclusions:**

The findings suggest that TRG serves as an independent prognostic factor for ESCC following NCRT. High GPR56 expression is found to be associated with a poor prognosis of ESCC. Downregulation of GPR56 suggests a potential significant predictive value in conjunction with MPR analysis.

## 1 Introduction

Esophageal cancer (EC) is one of the most common invasive malignancies worldwide. Recent data from 2020 underscores its substantial impact, ranking EC as the sixth leading cause of cancer-related deaths, with a staggering 600,000 new cases reported worldwide (1). Characterized by high incidence and mortality of EC (2), China is positioned as a country at increased risk, ranking seventh in incidence and fifth in mortality nationally according to the latest data from the National Cancer Center (3). Squamous cell carcinoma is the predominant histologic type, accounting for approximately 90% of all EC cases in the Asian (4). Surgery remains the primary treatment for EC patients, but the survival rates after single surgical intervention are often lower than expected (5). Recent studies have shown that surgery after neoadjuvant chemoradiotherapy (NCRT) can prolong survival and improve the quality of life in EC patients (6), making it as a standard treatment option for locally advanced or resectable EC (7).

NCRT improves tumor pathological response and also reduces tumor staging, postoperative metastasis, and recurrence rates, ultimately leading to improved survival. Based on this theoretical foundation, researches of tumor regression had gradually extended to various malignancies. However, there is no consensus on the scoring systems for tumor regression grade (TRG), including Mandard, Ryan, CAP, and the Japanese Society for Esophageal Diseases (JSED) standards. Recent researches indicate that the majority of international pathologists use the AJCC/CAP scoring system, which has also been adopted by the Chinese Society of Clinical Oncology (CSCO) (8). Studies using different TRG standards generally support a significant correlation between the extent of pathologic regression and overall survival, as well as an association between pathological response and postoperative recurrence (9, 10). Despite the higher rates of pathological regression and improved survival with NCRT, there is no consensus on whether tumor regression is an independent prognostic factor (11).

Furthermore, aberrant expression of tumor-related genes is known to play a critical role in cancer occurrence, progression, and prognosis (12). However, except for programmed cell death factor-1, there is limited research on changes in gene expression and prognosis of EC after NCRT. Therefore, the search for sensitivity markers for early prediction of NCRT efficacy and prognosis in EC is of paramount importance.

This study aims to validate the relationship between TRG and clinicalpathologic characteristics and the prognosis of esophageal squamous cell carcinoma (ESCC) following NCRT. Using bioinformatics methods, we identified key genes potentially associated with NCRT in ESCC. These genes will be validated for their expression changes before and after NCRT using immunohistochemical techniques. Subsequently, an exploration of their prognostic value will be investigated, providing potential molecular targets for prognostic assessment in ESCC.

## 2 Materials and methods

### 2.1 Data source

**Figure 1:**
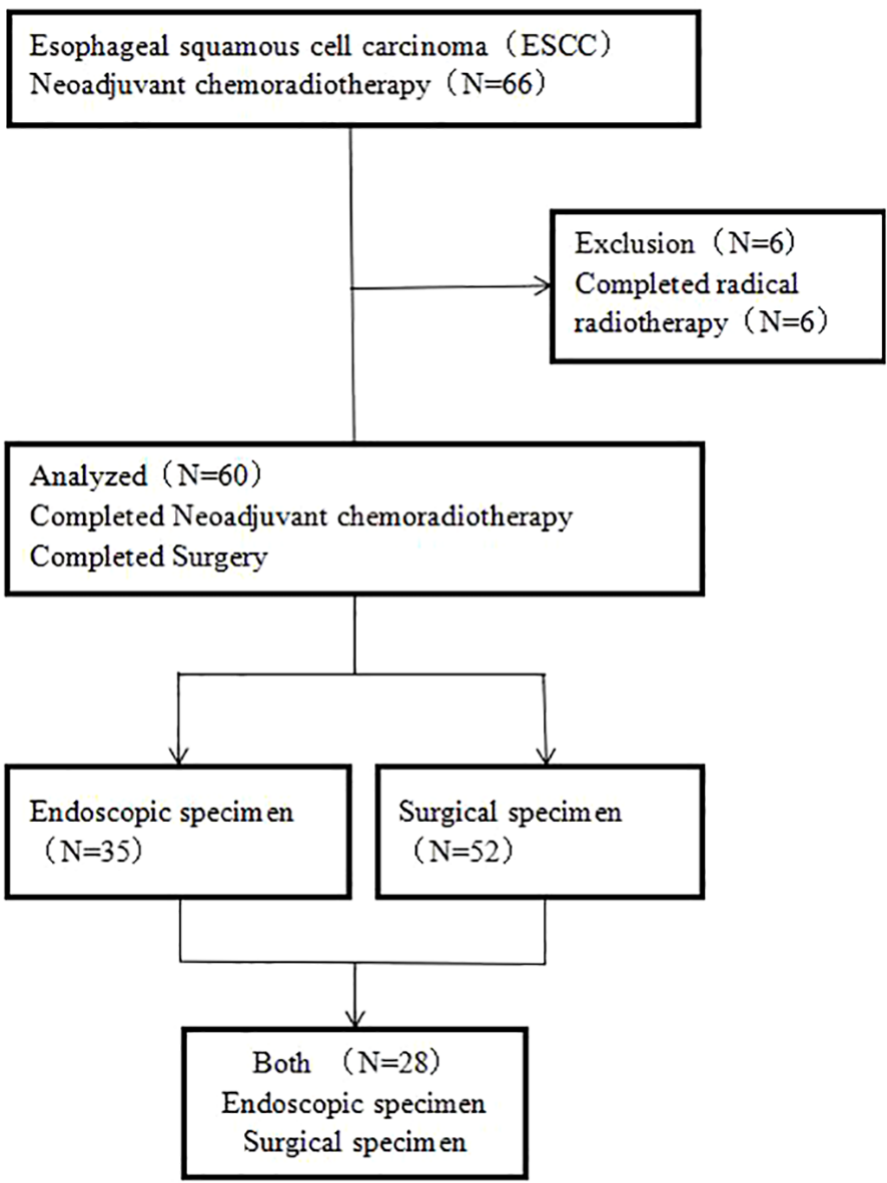
Inclusion patient selection flowchart.

This study is a retrospective analysis of ESCC patients who underwent surgery after NCRT at the People's Hospital Affiliated to Jiangsu University between January 2016 and February 2023. The screening process for enrolled patients is shown in **Figure 1**. 66 patients were initially selected and their medical histories were reviewed. Finally, 60 patients who met the criteria for completion of surgery after NCRT were included. Twenty-eight patients with pathologically confirmed ESCC were selected from pathology archives for endoscopic and surgical matched specimens. Inclusion criteria were as follows: (1) Age between 18 and 80 years; (2) Pathologic diagnosis of ESCC; (3) Completion of preoperative NCRT and at least one cycle of concurrent chemotherapy; (4) Tumor staging according to the 8th edition of the American Joint Committee on Cancer (AJCC) guidelines: cT1b~cT2 N+ or cT3~cT4a, any N staging; (5) General good health with normal cardiac, pulmonary, renal, and hematological functions; (6) Availability of complete medical records. Exclusion criteria were as follows: (1) Inability to tolerate surgery due to advanced age or other reasons, or presence of surgical contraindications; (2) Presence of other malignancies in addition to ESCC. As this was a retrospective analysis, informed consent was not required.

### 2.2 Methods

#### 2.2.1 Neoadjuvant chemoradiotherapy

**Radiotherapy**: The radiation target area included primary gross target volume (GTV-p), which consisted of the primary esophageal tumor and positive lymph nodes. Positive lymph nodes were defined as nodes visible on CT with a diameter of ≥1cm, showing marked enhancement on imaging regardless of size, or being detectable by ultrasound or endoscopic ultrasound, accompanied by disrupted dermal medullary structures. The primary clinical target volume (CTV-p) included a 3 cm external extension in both proximal and distal directions from the primary lesion toward the esophagus, with no expansion in anterior, posterior, left, or right directions. In the presence of metastatic lymph nodes (defined as gross target volume-node, GTV-N), the planning target volume (PTV) extended 1cm beyond the CTV-p and GTV-N. The total radiation dose was 41.4Gy/23FX delivered at 1.8Gy per fraction.

**Chemotherapy**: Most chemotherapy regimens consisted of platinum-based dual-drug combinations [cisplatin (75 mg/m^2^)/nedaplatin (80 mg/m^2^) and paclitaxel (175 mg/m^2^), 1 to 2 cycles at least, every 3 weeks].

**Surgical treatment**: Defined as radical resection surgery for EC, performed 4-8 weeks after NCRT.

**Surgical technique details**:

**Minimally invasive esophagectomy (MIE)**

**Figure 2:**
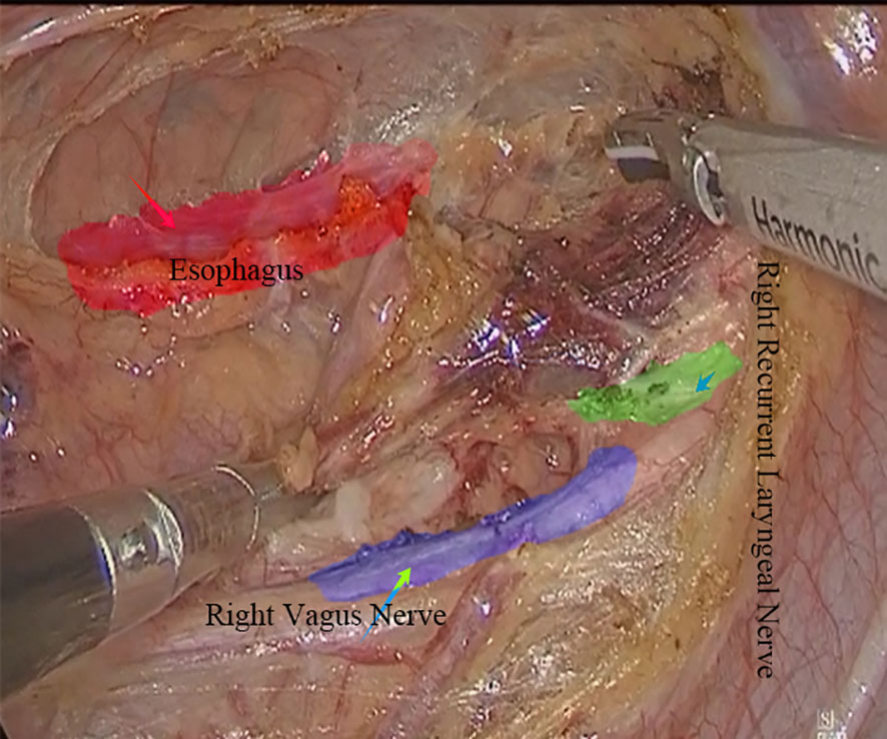
Minimally invasive esophagectomy.

Place the patient in the lateral position and mark the right mid-axillary line at the 8th intercostal for the observation port. An artificial pneumothorax was created with CO2 insufflation at a pressure of 10-12 mmHg. Under thoracoscopic guidance, surgical and auxiliary surgical ports were placed in the 3rd and 6th intercostal spaces on the midaxillary line and in the 6th intercostal space on the subscapularis angular line. With the assistance of thoracoscopy, the umbilical vein arch was ligated using vascular clips and ultrasonic knife, the esophageal lesion was mobilized. Thoracic duct ligation was performed, and lymph nodes were cleared adjacent to the esophagus, carinae area, and right and left recurrent laryngeal nerves. Negative-pressure drains were placed in the mediastinal and thoracic. Subsequently, in the supine position, an observation hole was made approximately 1.0 cm below the navel to establish an artificial pneumoperitoneum. Surgical ports were created on both the right and left sides of the navel, adjacent to the rectus abdominis muscle and below the rib arch along the midclavicular line. The stomach was mobilized, and the abdominal lymphadenectomy was performed. Under laparoscopic guidance, a tubular stomach was created from the lesser curvature. An incision was made at the anterior border of the left sternocleidomastoid muscle and an esophagogastric anastomosis was performed (**Figure 2**).

**Robotic-assisted minimally invasive esophagectomy(RAMIE)**

**Figure 3:**
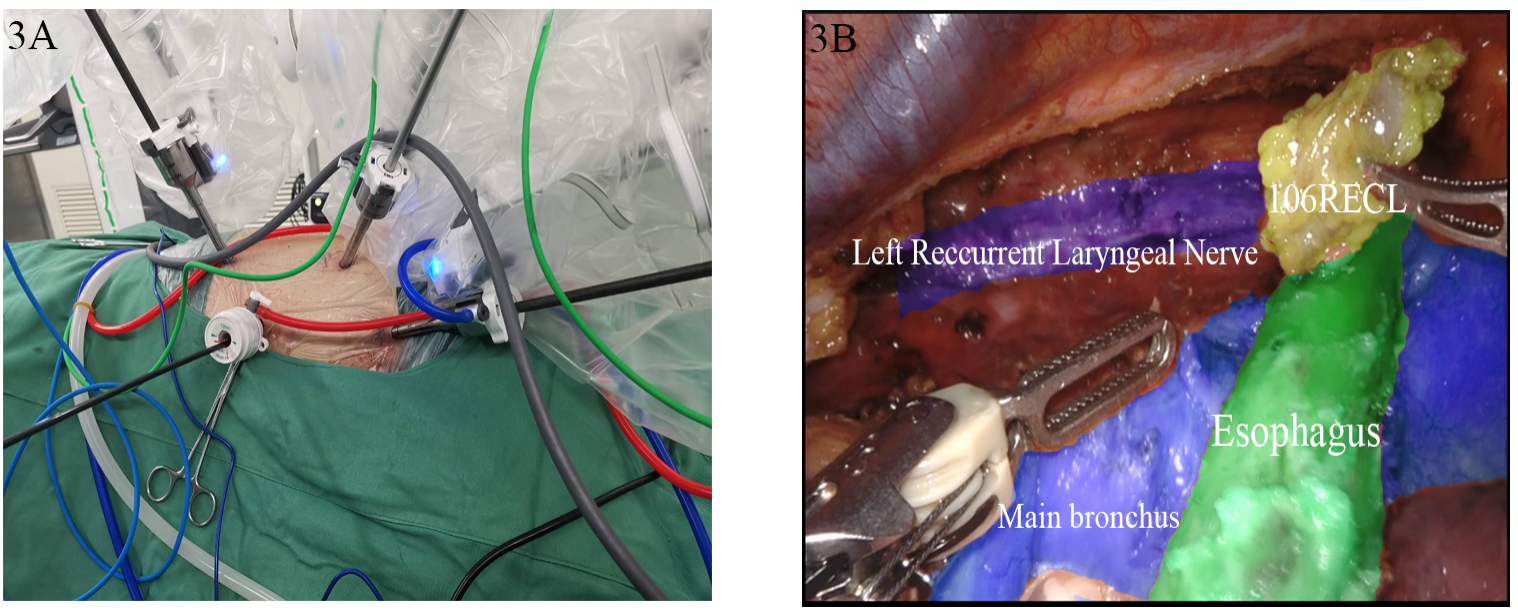
Robot-assisted minimally invasive esophagectomy. **(A)** External field of view; **(B)** Microscopic field of view.

In the lateral position, an 8 mm incision between the eighth intercostal space along the right midaxillary line was marked and selected preoperatively for lens placement. An 8-mm incision between the third intercostal space in the anterior axillary line was used as the fourth arm port, while a 12 mm incision at the sixth intercostal space on the anterior axillary line was used as the auxiliary operating port. An 8 mm incision at the sixth intercostal space along the subscapular line was used as the third entrance. The second robotic arm was inserted through the original entry site, the intrathoracic esophagus was mobilized, mediastinal lymphadenectomy was performed, and mediastinal and thoracic negative pressure drains were placed. In the supine position, an 8-mm Trocar was placed 2 cm to the right of the superior border of the navel as the second arm port, and an additional port was positioned at the right rectus abdominis muscle for the third arm. The original scope port was used as the fourth arm port to establish an artificial pneumoperitoneum. A 12 mm and a 5 mm Torcar were placed adjacent to the rectus abdominis muscle 2 cm below the rib arch along the midclavicular line as the auxiliary operating ports. The stomach was mobilized and the abdominal lymphadenectomy was performed with an ultrasonic scalpel. An incision was made at the anterior border of the left sternocleidomastoid muscle and an esophagogastric anastomosis was performed with instrumentation (**Figure 3**).

#### 2.2.2 Immunohistochemistry

For the 28 patients with paraffin-embedded specimens, immunohistochemical analysis was performed on endoscopically and surgically matched specimens. Rabbit monoclonal antibodies against MMP9 (1:1000, AB76003, Abcam), GPR56 (1:250, Ab302909, Abcam), and NFIX (1:200, PA564917, Thermo) were used in the immunohistochemistry. The Histo-score (H-score) method was used to interpret the results, with staining intensity categorized into four levels: 0 (negative), 1 (weak staining), 2 (moderate staining), and 3 (strong staining). The H-score was calculated using the following formula: H-score = (percentage of cells with no staining × 0) + (percentage of cells with weak staining × 1) + (percentage of cells with moderate staining × 2) + (percentage of cells with strong staining × 3). The H-score ranged from 0 (100% negative cells) to 300 (100% highly stained cells) (13). Each stained slide was independently reviewed and scored by two experienced pathologists. According to CAP standards: TRG 0 = no viable cancer cells (complete response); TRG 1 = single or small clusters of residual cancer cells (moderate response); TRG 2 = residual cancer with fibrosis in the stroma (mild response); TRG 3 = minimal or no tumor regression changes; large amounts of cancer cells remain (poor response).

#### 2.2.3 Definitions

Pathologic complete response(pCR) was defined as the lack of viable tumor cells in both the primary and metastatic lymph nodes. In contrast, MPR was defined as ≤10% of viable tumor cells in the original tumor bed, regardless of whether viable tumor cells were present in the metastatic lymph nodes. The pathological TRG was based on the classification standard of the American Joint Committee on Cancer/College of American Pathologists (AJCC/CAP) system. ypTNM stage was defined as pathological TNM stage after receiving neoadjuvant treatment. OS was defined as the time from the date of disease diagnosis to the date of death due to any cause or the date of the last follow-up for those alive.

#### 2.2.4 Gene chip data acquisition and analysis

The gene expression profile chip GSE43519, containing total RNA samples of peripheral blood from 21 paired ESCC patients before and after NCRT, was selected from the GEO database. Differential expressed genes were screened using R language with criteria of adj. *P* < 0.05 and |logFC| ≥ 1, and a volcano plot was generated using the ggplot 2 package.

#### 2.2.5 Statistical methods

Statistical analyses were performed using SPSS 25.0 software. Given the ordinal nature of TRG, ordered logistic analysis was used to assess the correlation between TRG and various clinicalpathologic features. Median with interquartile range (IQR) was used for continuous variables, and Fisher's exact probability test was used for categorical data. Kaplan-Meier methods and Log-Rank tests were used for survival analysis, and Cox proportional hazards regression models were used to evaluate OS. A significance level of *P* < 0.05 was considered statistically significant.

## 3 Results

### 3.1 Clinical data of enrolled patients

A total of 60 patients with EC were enrolled in this study, including 38 men (63.3%) and 22 women (36.7%), with a median age of 65 years (range, 50 to 77 years). 6 patients refused surgery after completing NCRT, and 60 patients who underwent surgery after NCRT. The majority of tumors were located in the middle-lower segment of the esophagus (n=53, 88.3%). Most patients had histologic types of moderately to well-differentiated squamous cell carcinoma (n=37, 61.7%). The majority of patients had tumors that did not involve nerves or vessels (n=55, 91.7%). Details of patient characteristics were shown in **Table 1**. The median operative time was 337 minutes (range: 164-890 minutes), and the median estimated blood loss was 100 mL (range: 20-500 mL). Surgical details were shown in **Table 2**. Postoperative surgical complications occurred in 21 patients (35.0%), with pulmonary complications occurred in 10 patients (16.7%) and anastomotic leakage occurred in 4 patients (6.7%). The median hospital stay was 21 days. Postoperative details were shown in **Table 3**.

**Table 1:** Clinical and pathologic characteristics of the study cohort.

Clinical data and pathological features	N (%)
Gender	
Male	38 (63.3%)
Female	22 (36.7%)
Age	
≥60 years	45 (75.0%)
<60 years	15 (25.0%)
BMI (m²/kg, X ± S)	22.63 ± 2.96
Length of tumor (cm, X ± S)	5.78 ± 2.19
Tumor location	
Upper	7 (11.7%)
Middle	25 (41.7%)
Lower	28 (46.7%)
Differentiation degree	
Poorly to undifferentiated	23 (38.3%)
Moderately to highly differentiated	37 (61.7%)
Histological Type	
Medullary	18 (30.0%)
Fungating	8 (13.3%)
Ulcerative	20 (33.3%)
Constrictive	14 (23.3%)
Neural or vascular invasion	
Absent	55 (91.7%)
Present	5 (8.3%)
yp T stage	
T0	27 (45.0%)
T1	8 (13.3%)
T2	10 (16.7%)
T3	15(25.0%)
yp N stage	
N0	43 (71.7%)
N1	11 (18.3%)
N2	4 (6.7%)
N3	2 (3.3%)

BMI, body mass index; TNM, tumor-node-metastasis.

**Table 2:** Operative details (n = 60).

Time of surgery, median (sd), minutes	337 (106)
Intraoperative blood loss, median (sd), mL	123 (82)
Conversion to open, no. (%)	1 (1.7%)
Intraoperative complications, no. (%)	0 (0)
Radicality of surgery	
R0, no. (%) R1, no. (%)	60 (100%) 0 (0)
Types of surgery	
OE, no. (%) MIE, no. (%) RAMIE, no. (%)	20 (33.3%) 38 (63.3%) 2 (3.3%)

OE, open esophagectomy; MIE, minimally invasive esophagectomy; RAMIE, robotic-assisted minimally invasive esophagectomy; SD, standard deviation.

**Table 3:** Postoperative details (n = 60).

Length of Stay, days (median - range)	21 (16-104)
Overall complications, no. (%)	21 (35.0%)
Pulmonary complications, no. (%)	10 (16.7%)
Pneumonia, no. (%)	6 (10.0%)
Pneumothorax, no. (%)	2 (3.3%)
Pleural effusion, no. (%)	2 (3.3%)
ARDS, no. (%)	0 (0)
Cardiac complications, no. (%)	0 (0)
Thoracic empyema, no. (%)	1 (1.7%)
Anastomotic leakage, no. (%)	4 (6.7%)
Tracheoesophageal fistula, no. (%)	1 (1.7%)
Chylothorax, no. (%)	1 (1.7%)
Wound infection, no. (%)	4 (6.7%)
30-day mortality, no. (%)	0 (0)
60-day mortality, no. (%)	0 (0)
90-day mortality, no. (%)	0 (0)
Resected lymph nodes, no. (median - range)	13 (11-31)

### 3.2 Analysis of factors influencing TRG and clinical pathologic characteristics

According to histopathologic grading of CAP, TRG 0 (complete pathologic response) was the most common histologic response (n=21, 35.0%), followed by TRG 3 (n=17, 28.3%), TRG 1 (n=15, 25.0%), and TRG 2 (n=7, 11.7%). The relationships between TRG and preoperative clinical and pathologic characteristics were shown in **Table 4**. There were no statistically significant associations between TRG and gender, age, length of tumor, clinical T stage, clinical N stage, or clinical TNM stage. Poor to undifferentiated tumors and the presence of neural or vascular invasion were associated with inadequate TRG, and showed significant differences. Multivariate analyses identified tumor differentiation and the presence of neural or vascular invasion as independent predictors of TRG.

**Table 4:** Logistic analysis of TRG and clinical pathologic characteristics.

Variables	TRG 0	TRG 1	TRG 2	TRG 3	Univariate	*P*	Multivariable	*P*
					OR (95% CI)		OR (95% CI)	
Gender								
Male	13	10	3	12	1.149 (0.444-2.975)	0.774		
Female	8	5	4	5	Ref.	-		
Age								
≥60	16	14	5	10	0.465 (0.159-1.358)	0.162		
<60	5	1	2	7	Ref.	-		
Length of tumor (cm)	5.71 ± 2.35	6.00 ± 2.17	6.00 ± 2.77	5.56 ± 1.91	0.987 (0.800-1.219)	0.907		
Tumor differentiation								
Moderately to highly differentiated	7	12	5	13	Ref.	-	Ref.	-
Poorly to undifferentiated	14	3	2	4	0.229 (0.083-0.633)	0.005	0.180 (0.062-0.529)	0.002
Neural/Vascular invasion								
Positive	0	1	0	4	Ref.	-	Ref.	-
Negative	21	14	7	13	0.081 (0.009-0.755)	0.027	0.053 (0.005-0.556)	0.014
Constricted Type	4	4	2	4	Ref.	-		
Clinical T category								
T2	1	1	1	0	0.462 (0.034-6.320)	0.563		
T3	20	11	5	16	0.629 (0.119-3.325)	0.585		
T4	0	3	1	1	Ref.	-		
Clinical N category								
N0	8	3	2	7	1.490 (0.162-13.713)	0.725		
N1	9	8	3	10	1.731 (0.197-15.200)	0.621		
N2	3	3	1	0	0.685 (0.056-8.356)	0.767		
N3	1	1	1	0	Ref.	-		
Clinical TNM stage								
II	8	3	2	6	0.492 (0.115-2.094)	0.337		
III	12	9	3	8	0.453 (0.117-1.757)	0.252		
IV	1	3	2	3	Ref.	-		

TRG, tumor regression grade; TNM, tumor-node-metastasis; OR, odds ratio; CI, confidence interval; Ref, reference.

### 3.3 Analysis of TRG and perioperative factors

All patients who underwent radical esophagectomy after NCRT achieved R0 resection. The rate of pCR after completion of NCRT was 35.0%, with an MPR reaching 60.0%. There were 27 cases (45.0%) in which the primary tumor achieved ypT0, including 6 patients (10.0%) with lymph node metastasis (ypT0N+). The postoperative distribution of yp stages included stage I (n=32, 53.3%), stage II (n=11, 18.3%), stage III (n=15, 25.0%), and stage IV (n=2, 3.3%). TRG grading was significantly associated with lymph node involvement (*P* < 0.05), ypT staging (*P* < 0.001), ypN staging (*P* < 0.05), and ypTNM staging (*P* < 0.001), as shown in **Table 5**.

**Table 5:** Relationship between TRG and postoperative pathologic factors.

Variables	TRG 0	TRG 1	TRG 2	TRG 3	Univariate OR (95% CI)	*P*
Postoperative Complications						
Yes	3	3	2	2	Ref.	–
No	18	12	5	15	1.012 (0.296-3.456)	0.985
Lymph node Total count	11.90 ± 5.21	15.00 ± 5.31	9.29 ± 3.86	14.35 ± 8.49	1.033 (0.960-1.111)	0.390
Positive Lymph node Count	0	0.87 ± 1.06	0.43 ± 0.54	1.65 ± 2.62	1.940 (1.162-3.237)	0.011
ypT stage						
T0	21	5	0	1	0.006 (0.001-0.053)	< 0.001
T1-3	0	10	7	16	Ref.	–
ypN stage						
N0	21	6	6	10	Ref.	–
N1-3	0	9	1	7	3.432 (1.191-9.889)	0.022
ypTNM Stage						
I	21	2	4	5	0.113 (0.039-0.327)	< 0.001
II-IV	0	13	3	12	Ref.	–

TRG, tumor regression grade; TNM, tumor-node-metastasis; OR, odds ratio; CI, confidence interval; Ref, reference.

### 3.4 Impact of tumor regression on patient prognosis

**Figure 4:**
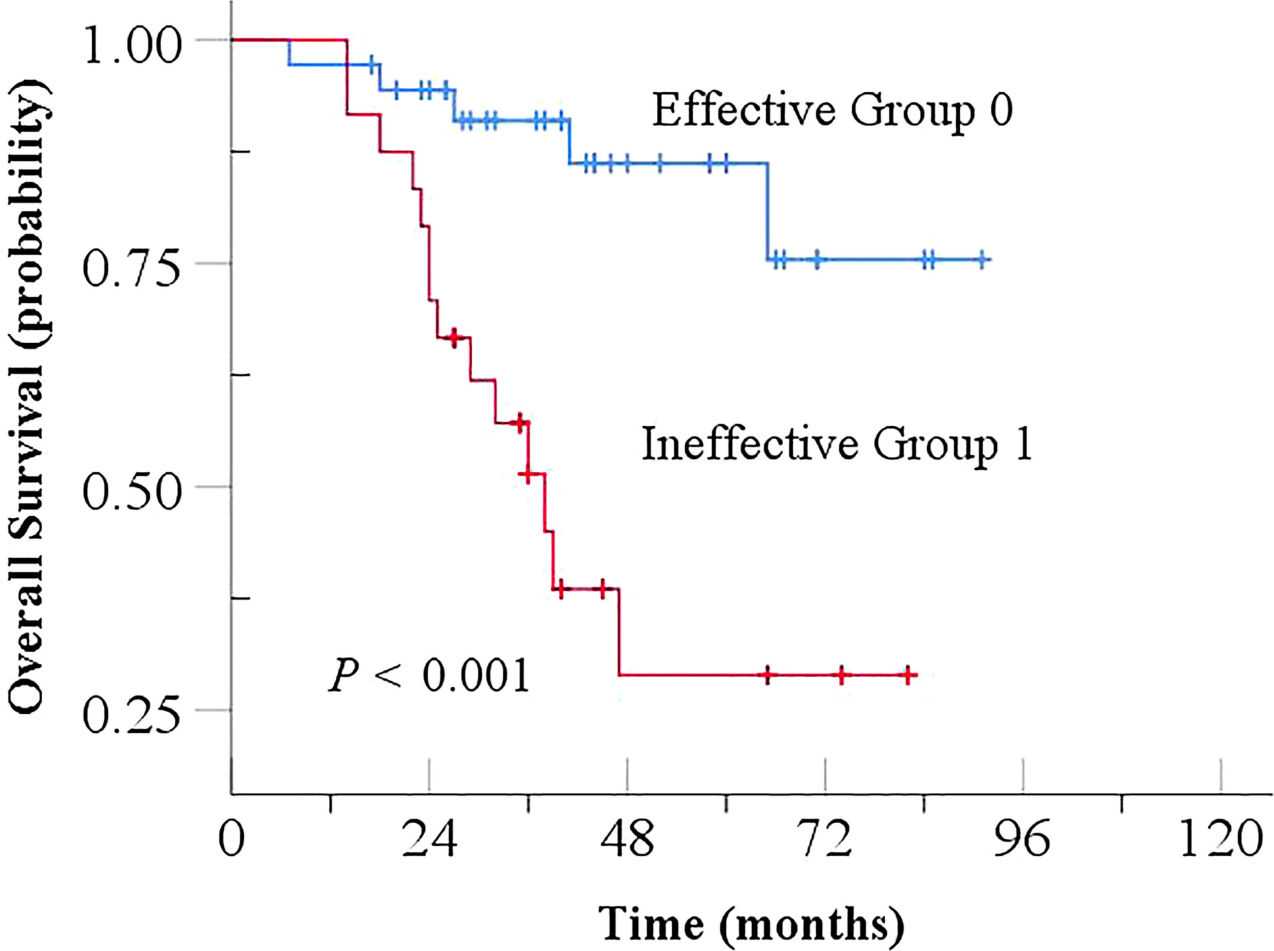
Kaplan-Meier curve for Overall Survival based on TRG in NCRT. TRG, tumor regression grade; NCRT, neoadjuvant chemoradiotherapy.

In the study, the effectiveness of NCRT for EC was stratified into an effective group (TRG 0 + 1) and an ineffective group (TRG 2 + 3) based on TRG values. The effective group exhibited a significantly longer OS (Log-Rank, *P* < 0.001), indicating a strong association between lower TRG and prolonged OS, as shown in **Figure 4**. Univariate analyses showed that gender, age, tumor length, ypN stage were not associated with outcome. However, pathologic differentiation, pCR, ypT stage and TRG were significantly correlated with prognosis (*P* < 0.05). Further Cox multivariate analysis, including parameters with *P* < 0.05, identified TRG as an independent prognostic factor for OS (HR: 4.271, *P* = 0.021, 95% CI 1.242-14.692), as shown in **Table 6**.

**Table 6:** Cox regression analysis of OS in patients undergoing NCRT.

Variables	ref/	Univariate HR (95%CI)	*P*	Multivariate HR (95%CI)	*P*
Gender	Male/Female	0.927 (0.364-2.361)	0.874		
Age	≥60/<60	1.457 (0.552-3.846)	0.447		
Tumor length (cm)	<4/≥4	3.303 (0.440-24.777)	0.245		
Histologic grade	Undifferentiated-low/Moderate-high	1.585 (0.362-6.939)	0.038	3.084 (0.686-13.869)	0.142
ypT stage	T0/T1-3	3.440 (1.139-10.386)	0.028	1.977 (0.502-7.786)	0.330
ypN stage	N0/N1-3	2.305 (0.918-5.789)	0.075		
pCR	NO/YES	0.089 (0.012-0.671)	0.019	0.191 (0.018-2.064)	0.173
TRG	0+1/2+3	5.600 (1.996-15.707)	0.001	4.271 (1.242-14.692)	0.021

TRG, tumor regression grade; TNM, tumor-node-metastasis; HR, hazard ratio; CI, confidence interval; Ref, reference; OS, Overall Survival; NCRT, neoadjuvant chemoradiotherapy; pCR, pathologic complete response.

### 3.5 Differential gene expression of ESCC before and after NCRT

**Figure 5:**
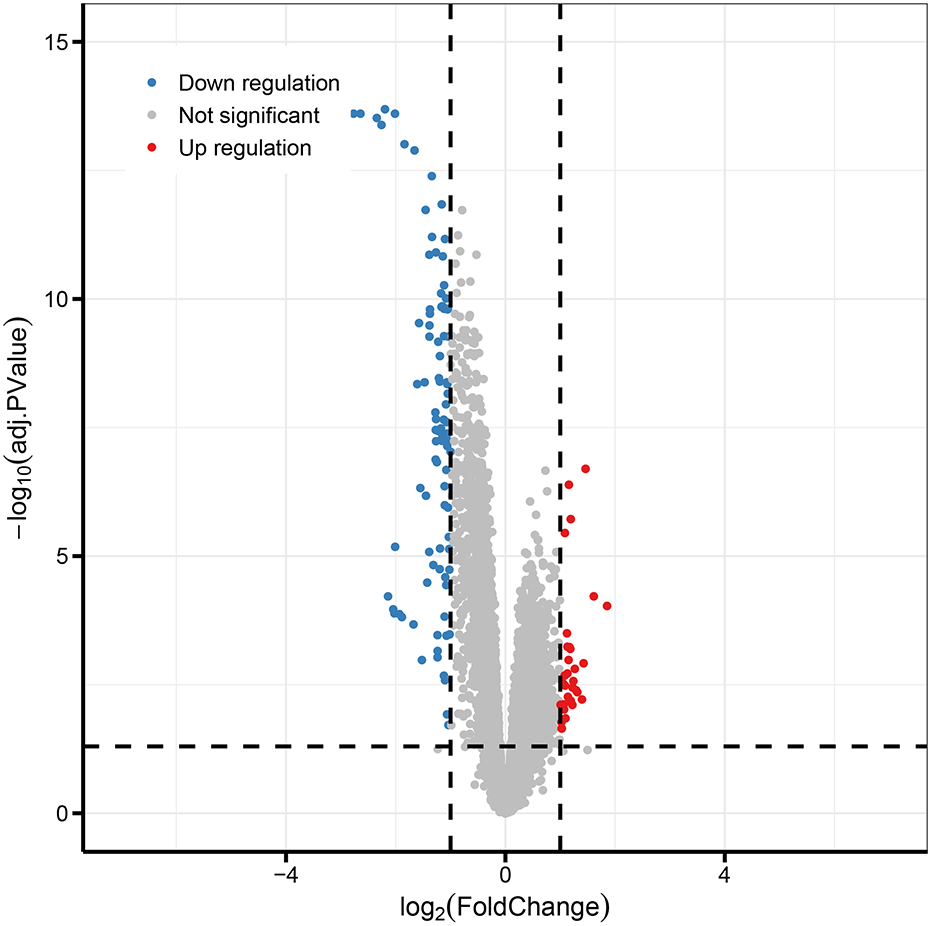
Volcano plot of differentially expressed genes.

**Figure 6:**
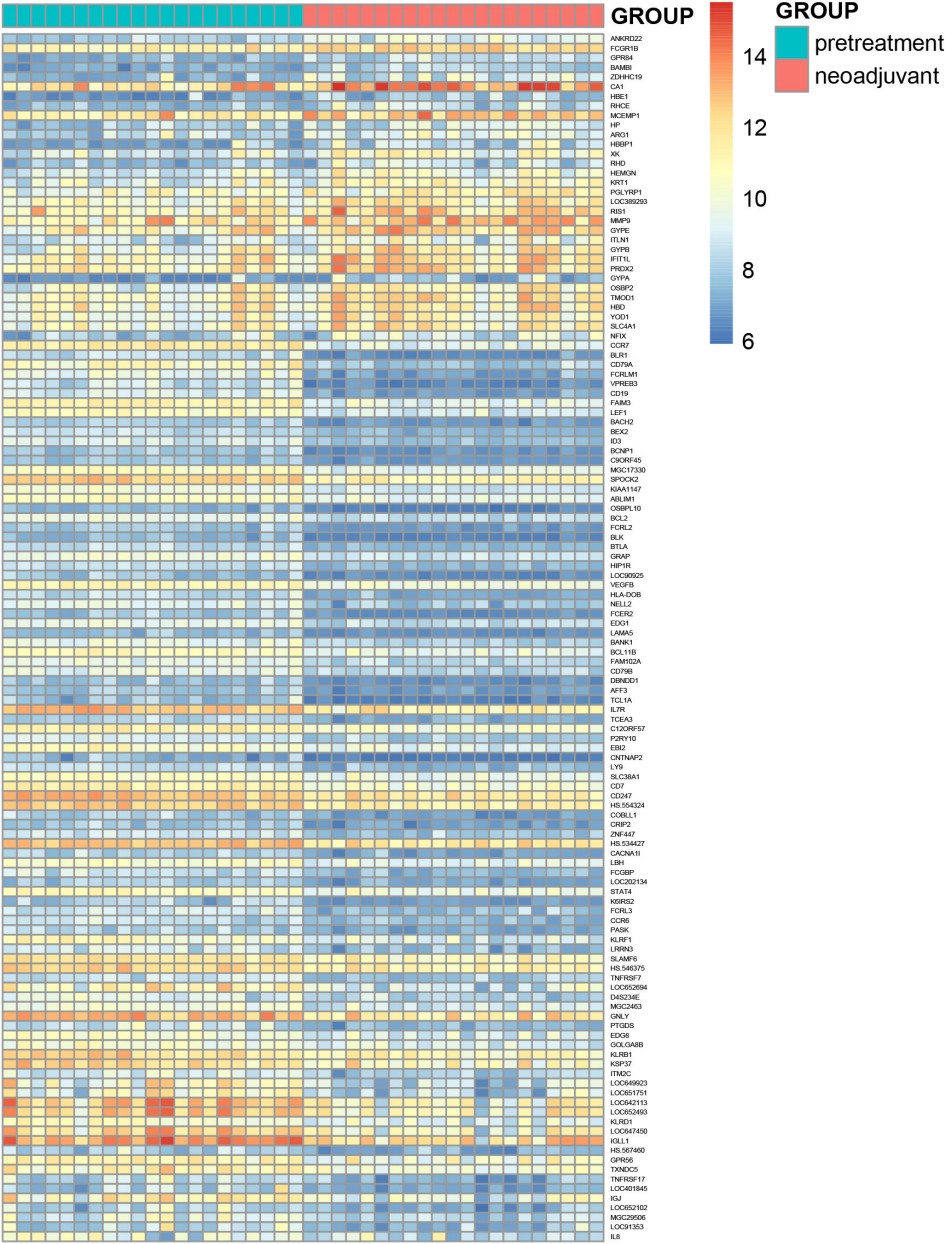
Hierarchical clustering heatmap of differentially expressed genes between two groups.

The gene expression profile chip GSE43519, representing ESCC before and after NCRT, was selected for analysis. Using the statistical programming language R, a filtering criterion of adj. *P* < 0.05 and |logFC| ≥ 1 was applied. A total of 126 differentially expressed genes were identified, including 32 upregulated genes and 94 downregulated genes (**Supplementary Table S1**). Volcano plots and heatmaps were generated based on the identified differentially expressed genes (**Figures 5**, **6**).

(The horizontal axis represents the fold change in gene expression, while the vertical axis represents the significance of the differences. Red dots indicate upregulated genes, blue dots indicate downregulated genes, and gray dots indicate genes with no significant differences.)

### 3.6 Expression of MMP9, NFIX, and GPR56 in ESCC tissues

**Figure 7:**
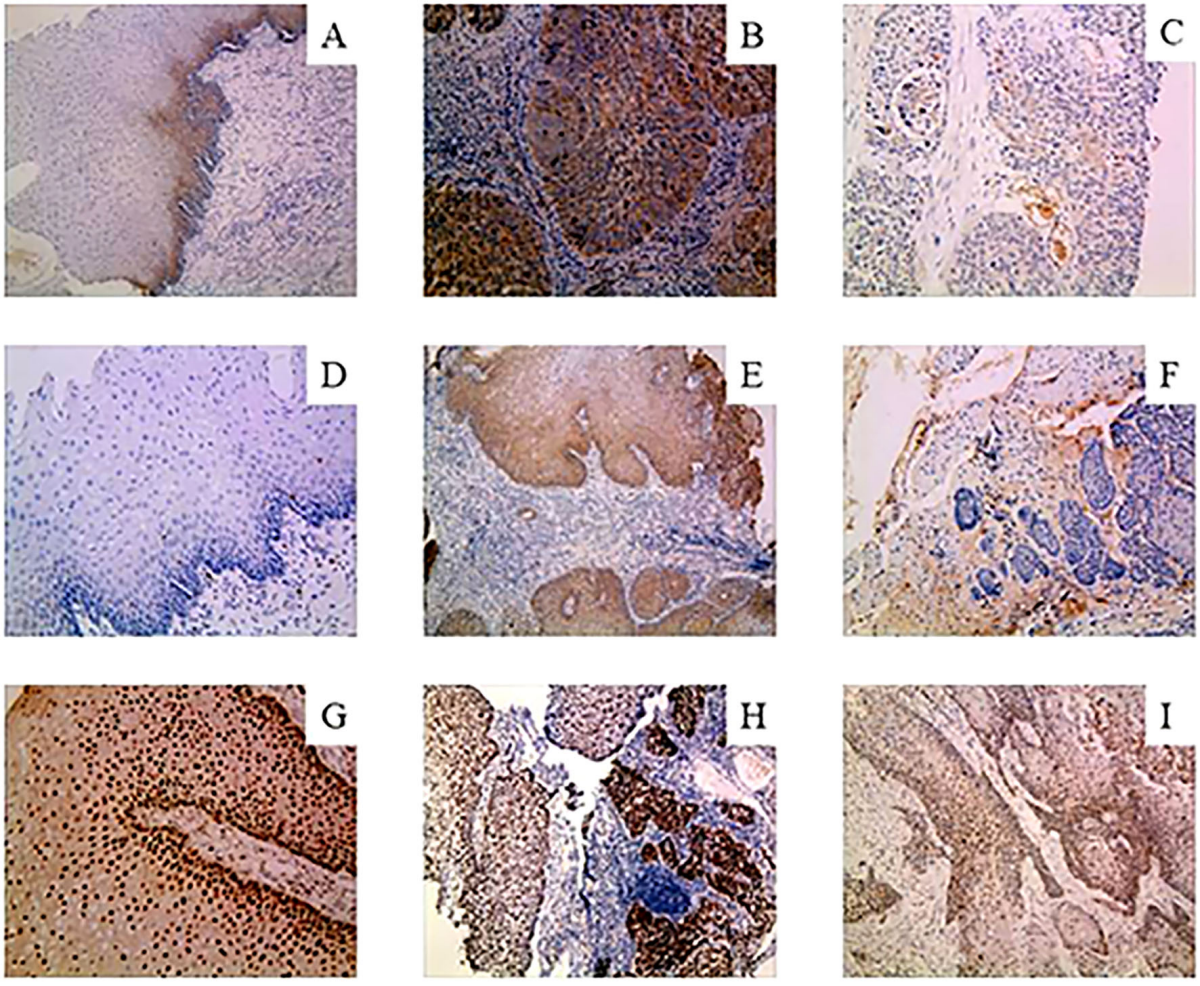
Immunohistochemical staining for GPR56, MMP9, and NFIX. **(A-C)**, Immunohistochemical images of GPR56 in paired paracancer tissue, pre-NCRT and post-NCRT esophageal cancer samples. **(D-F)**, Immunohistochemical images of MMP9 in paired paracancer tissue, pre-NCRT and post-NCRT esophageal cancer samples. **(G-I)**, Immunohistochemical images of NFIX in paired paracancer tissue, pre-NCRT and post-NCRT esophageal cancer samples. NCRT, neoadjuvant chemoradiotherapy.

**Figure 8:**
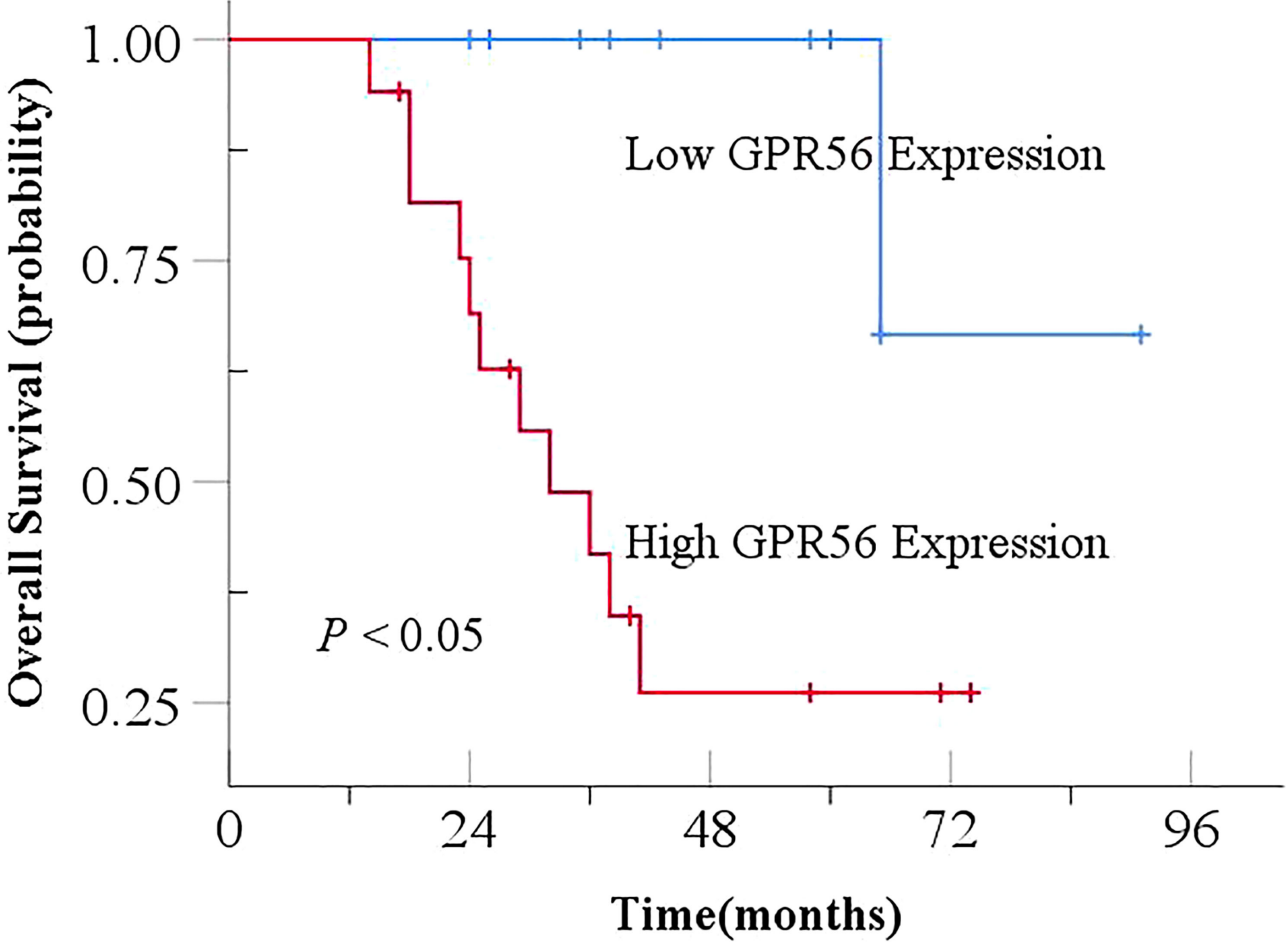
Effect of GPR56 expression on overall survival in ESCC. ESCC, Esophageal squamous cell carcinoma.

Based on the existing literature, we selected the genes of interest MMP9, NFIX and GPR56 from the differentially expressed genes for further analysis. Immunohistochemical staining showed that positive NFIX expression was localized in the nucleus of cells, while MMP9 and GPR56 showed positive staining in the cell membrane and cytoplasm of cells, as shown in **Figure 7**. Immunohistochemistry results showed that the positivity rate of GPR56 and MMP9 was higher than those in paracancerous tissues (*P* < 0.05), and the positivity rate of NFIX was lower than that in paracancerous tissues, but there was no statistically significant difference (*P* > 0.05). The positivity rates of GPR56, MMP9, and NFIX in tissues after NCRT were not significantly different from those in paracancerous tissues (**Table 7**). GPR56 expression in ESCC tissues was divided into low and high expression groups by Histo-score criterion (using Log-Rank survival analysis with the H-score at the minimum *P*-value as the optimal cutoff). The analysis showed a significant reduction in the expression of GPR56, MMP9, and NFIX after NCRT (*P* < 0.001, **Table 8**). Kaplan-Meier survival analysis suggested a significantly shortened OS in ESCC patients with high GPR56 expression (*P* < 0.05, **Figure 8**).

**Table 7:** Comparison of the expression of GPR56, MMP9, and NFIX in tissues.

Variables	GPR56	MMP9	NFIX		GPR56	MMP9	NFIX
Cancer	22 (78.5)	20 (71.4)	15 (53.6)	Post-NCRT	13 (46.4)	10 (35.7)	18 (64.3)
Paracancer	8 (28.6)	8 (28.6)	21 (75.0)	Paracancer	8 (28.6)	8 (28.6)	21 (75.0)
χ^2^	14.072	10.286	2.800		1.905	0.327	0.760
*P*	0.000	0.001	0.094		0.168	0.567	0.383

NCRT, neoadjuvant chemoradiotherapy.

**Table 8:** Correlation of gene expression in neoadjuvant chemoradiotherapy.

	H-score (pre-NCRT)	H-score (post-NCRT)	t	*P*
GPR56	109.29 ± 51.20	22.32 ± 34.68	8.504	<0.001
MMP9	82.14 ± 69.09	32.86 ± 50.98	4.418	<0.001
NFIX	119.29 ± 41.89	43.57 ± 38.51	9.454	<0.001

NCRT, neoadjuvant chemoradiotherapy.

### 3.7 Relationship between the expression of GPR56 and prognosis after NCRT

**Figure 9:**
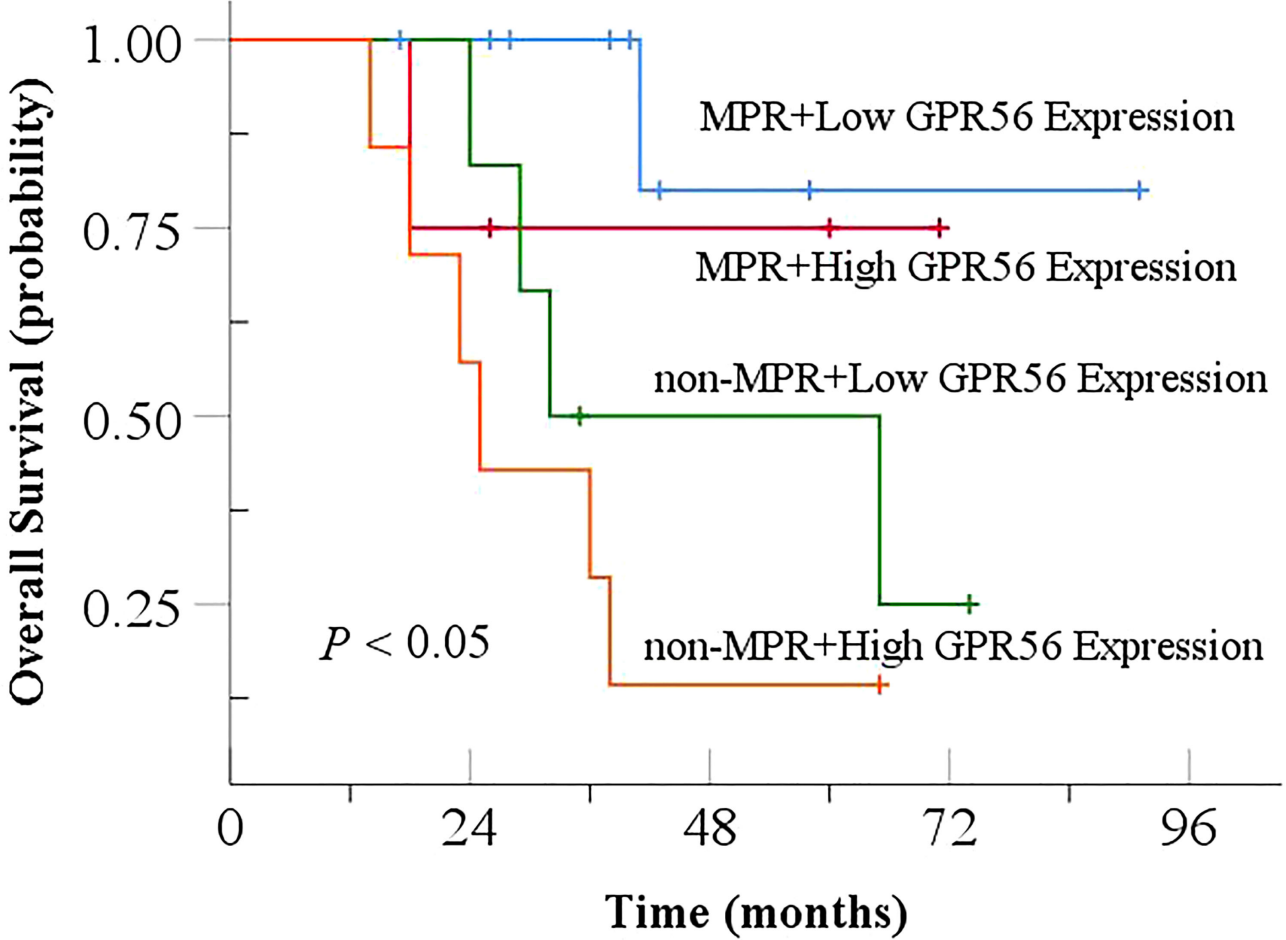
Relationship between GPR56 gene expression and MPR in combination analysis with Overall Survival after NCRT. NCRT, neoadjuvant chemoradiotherapy; MPR, major pathological response.

Among the 28 patients with both pre- and post-NCRT specimens, 14 patients achieved MPR after treatment (14/28, 50%), and the remaining 14 patients did not achieve MPR (non-MPR, 14/28, 50%). As shown in **Figure 9**, patients achieving MPR with low GPR56 expression had the highest OS, followed by patients achieving MPR with high GPR56 expression and non-MPR patients with low GPR56 expression. Patients with non-MPR and high GPR56 expression had the worst OS (*P* < 0.05).

## 4 Discussion

Esophageal cancer is a common malignancy of gastrointestinal tract, with esophagectomy remaining the primary surgical treatment. However, open esophagectomy(OE) is associated with significant trauma, prolonged incision healing time, and a high incidence of complications. As a result, it has been gradually replaced by minimally invasive esophagectomy(MIE), which reduces surgical trauma, shortens incision healing time, and does not increase the risk of severe complications (14). In recent years, with the development of surgical technology, robot-assisted minimally invasive esophagectomy (RAMIE) has been used in the clinic, and Preliminary findings have demonstrated that RAMIE is feasible, safe, and effective in clinical practice (15, 16). This is similar to our findings that minimally invasive surgery reduces intraoperative bleeding and does not significantly prolong the duration of surgery or increase the risk of surgical complications compared with open surgery (**Supplementary Table S2**).

Neoadjuvant chemoradiotherapy(NCRT) is a common standard of treatment for locally advanced or resectable EC. NCRT aims to reduce tumor burden, decrease tumor activity, minimize intraoperative metastasis, and reduce postoperative recurrence rates, thereby improving the potential success of surgical resection for resectable EC. TRG is commonly used to assess the efficacy of NCRT, and there is evidence that it correlates with the prognosis of EC (17). This correlation has been demonstrated in studies such as the Dutch CROSS trial and the Chinese NEOCRTEC5010 trial (18, 19). NCRT not only increases the rate of complete resection but also improves the prognosis of ESCC patients. The NEOCRTEC5010 trial demonstrated a correlation between pathologic response and the prognosis in locally advanced ESCC (20). In the neoadjuvant treatment of ESCC, previous studies have shown that ESCC patients have favorable pathologic tissue responses regardless of whether radiotherapy is administered, and there is no significant difference in treatment-related adverse events (21). This is consistent with our previous research in which the occurrence of radiation pneumonitis after radiotherapy for ESCC was controllable, possibly by regulating radiation-related factors to minimize adverse effects (22). Based on our previous studies, the application of personalized treatment to control high-risk factors for radiation therapy-related adverse effects better ensured the treatment outcomes for the patients included in this study. In our current research, the completion rate of NCRT reached 100%, with pCR and MPR rates of 35.0% and 60.0%, respectively.

The TRG scoring system is critical for assessing the pathologic response of tumors. In 2019, Dr. Takeda and colleagues introduced the Ryan scoring system to assess the prognosis of NCRT in EC, and confirmed its correlation with OS (9). However, the three-tiered system of the Ryan scoring may have limitations in practical application in the current context of multimodal therapy. Therefore, researchers have investigated and proposed new TRG standards. The College of American Pathologists (CAP) has modified the Ryan scoring system, and established a four-tiered standard (23). CAP has become one of the most widely used TRG standards for EC internationally (8), and has been incorporated into the 2020 CSCO Esophageal Cancer Diagnosis and Treatment Guidelines. Compared with Dr. Takeda's research, we not only confirmed the correlation between CAP score and OS in ESCC but also further demonstrated that CAP score is an independent prognostic factor for ESCC. However, our study is limited to ESCC, and whether CAP score remains valuable in a cohort including both ESCC and esophageal adenocarcinoma (EAC) requires further investigation.

In addition, our study suggests an association between a good pathologic response and OS in ESCC patients. Currently, tumor-related genes play a critical role in regulating the processes of tumor occurrence and development, influencing patient prognosis. Therefore, in the research of NCRT for ESCC, we identified the tumor-related differentially expressed gene GPR56 based on results from public databases. As a member of the G protein-coupled receptor (GPCR) family, GPR56 is an adhesive receptor involved in signaling pathways related to cell proliferation, migration, and adhesion (24). Abnormal expression and gene mutations of GPR56 are closely associated with tumor proliferation, migration, and invasion, and show a negative correlation with the prognosis of various tumors (25). In ESCC, GPR56 is mainly characterized by overexpression, which is associated with metastasis and invasion (26, 27). In addition, LIM et al. (28) found a correlation between GPR56 expression and the prognosis in colorectal cancer. The high expression of GPR56 was associated with a higher TNM stage and a negative correlation with OS in colorectal cancer. This suggests that GPR56 may be closely related to the tumor prognosis. In this study, we not only evaluated the impact of GPR56 expression on the prognosis of ESCC patients, but also explored that the combined analysis of GPR56 expression with pathological response may have more significant predictive value for therapeutic efficacy. However, the biological functions and potential mechanisms of GPR56 in ESCC still require further investigation.

This study is a single-center, retrospective, observational study with limitations such as insufficient sample size and follow-up time. Potential selection bias must be considered. Therefore, further verification is needed by large-sample prospective controlled studies, including EAC.

## 5 Conclusion

Our study data indicate that the TRG according to the CAP scoring system after NCRT may serve as an independent prognostic factor for ESCC. It correlates with tumor differentiation and the presence of neural or vascular invasion. The sensitivity gene GPR56 in NCRT for ESCC showed a significant decrease in expression, and in combination with MPR analysis, it may have more significant predictive value for therapeutic efficacy, serving as a potential molecular marker for predicting the effectiveness of NCRT for ESCC.

## Data Availability

All datasets used and/or analyzed during the current study are available from the corresponding author on reasonable request.

## Supplementary material

Supplementary Table 1 Differential expression genes before and after NCRT of the ESCC.
