# Supplementary material for: Identification of biomarkers for tumor regression grade in esophageal squamous cell carcinoma patients after neoadjuvant chemoradiotherapy
Source: Front Oncol. 2025 Jan 17;14:1426592. doi: 10.3389/fonc.2024.1426592 (PMC11782036; doi:10.3389/fonc.2024.1426592)
Supplement: Supplementary file 2 [file Table2.docx]

**Supplementary table 2**. Operative details

|  | MIE | OE | t | *P* |
| --- | --- | --- | --- | --- |
| Time of surgery (min) | 346.13±76.56 | 329.00±148.80 | 0.418 | 0.677 |
| Intraoperative blood loss(ml) | 92.56±54.57 | 178.57±94.302 | -4.486 | <0.001 |
| Length of Stay(days) | 23.23±18.46 | 19.29±21.97 | 0.738 | 0.463 |

OE, open esophagectomy; MIE, minimally invasive esophagectomy
